# Identification of an unconventional process of instrumental learning characteristically initiated with outcome devaluation-insensitivity and generalized action selection

**DOI:** 10.1038/srep43307

**Published:** 2017-02-27

**Authors:** Yoshio Iguchi, Ziqiao Lin, Hiromi Nishikawa, Yoshio Minabe, Shigenobu Toda

**Affiliations:** 1 Department of Psychiatry & Behavioral Science, Kanazawa University School of Medicine, Kanazawa, Ishikawa, Japan; 2 Department of Molecular Genetics, Institute of Biomedical Sciences, Fukushima Medical University, Fukushima, Japan; 3 Research Center for Child Mental Development, Kanazawa University, Kanazawa, Ishikawa, Japan

## Abstract

The distinction between goal-directed action and habitual response, particularly with respect to moderate or extended appetitive instrumental training, is well documented; however, the propensity toward instrumental behavior in the early training stage has not been elucidated. In this study, we trained Sprague Dawley rats to press a lever to obtain food as an outcome for various time periods and monitored the changes in their sensitivity to outcome devaluation and choice between the levers they had been trained with and unfamiliar levers. After the extensive training with a random interval schedule, the rats were insensitive to outcome devaluation, and exhibited a typical habit-like phenotype, as previously reported, and the untrained leverpresses were relatively rare and sporadic. During the initial stage of training (≤1 week), the rats exhibited a similar insensitivity to the devaluation; however, in contrast to the overtrained condition, they performed distinctive unbiased leverpresses on both the trained and untrained levers. Thus, we propose a possibility that, contrary to the authentic concept that instrumental learning is initiated with an outcome devaluation-sensitive goal-directed stage, under some conditions, this learning can unconventionally begin with the initial stage that is distinct from both goal-directed action and habitual response.

Animals learn contingency between their own behavior and the consequent outcomes of their behavior, thereby allowing themselves to determine whether they should continue or abandon a behavior based on the motivation to gain a preferable outcome or to avoid an aversive outcome, respectively. Instrumental/operant learning with an appetitive outcome, such as food, water, or monetary reward, is considered as an elementary process of decision-making executed by animals in order to adapt to the complex contingencies in their environments^1^,^2^,^3^. Instrumental behavior has been understood to be initially goal-directed as its performance is highly sensitive to changes in the incentive value of outcomes and in the behavior-outcome contingencies for the moment-by-moment flexible optimization of actions that require energy and concentration^4^,^5^. As the operation is repeated extensively, the goal-directed action transforms into a habitual response, where its performance is converted from intentional to automatic, thereby facilitating prompt and highly optimized performance^6^. Thus, insensitivities to outcome devaluation and contingency degradation after extended practice are regarded as the major hallmarks of habit formation^7^,^8^.

The contrast between a goal-directed action and habitual response in appetitive instrumental learning has been investigated extensively^2^,^3^,^9^, but little is known about the behavioral characteristics of the earlier, initial stage. In particular, do goal-directed actions commence at the very start of instrumental learning^10^, or do other distinctive learning processes exist before goal-directed actions? If the latter is true, what is the difference between the initial process and those that follow? To address these questions, we trained rats to press a lever to obtain a food in order to analyze the changes in the sensitivity of the instrumental performance to outcome devaluation after 2, 8, 16, and 31 daily training sessions. We also examined the choices made by the animals with two simultaneously presented levers: one on which they were trained with reinforcement and another on which they never received training with reinforcement (unfamiliar lever), at each stage of the training. We then analyzed the number and temporal distribution of responses to the two levers to investigate whether the action choice was generalized toward the untrained lever or biased toward the trained lever^11^,^12^.

## Results

### Instrumental leverpress training proceeded steadily

**Figure 1:**
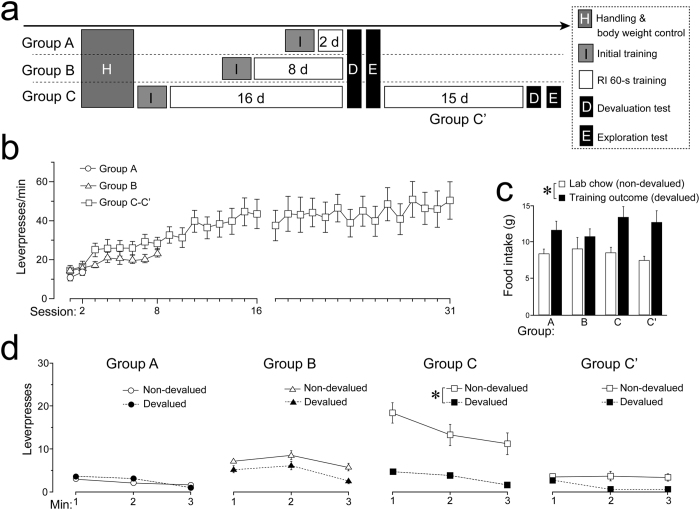
Sensitivity to outcome devaluation was exhibited in the middle but not the initial or late stages of instrumental training. (**a**) Timeline for the entire experimental procedure. Each group underwent assigned RI 60-s training sessions (Group A, 2 days, *n* = 11; Group B, 8 days, *n* = 11; and Group C, 16 days, *n* = 10), before undergoing outcome devaluation (box d) and two-lever tests (Fig. 2). For the rats in Group C, leverpress was retrained on an RI 60-s schedule for more 15 days (Group C’). Second devaluation and two-lever tests were conducted in the same manner as that of the first set. (**b**) Mean ( ± *SEM*) leverpresses per minute over RI 60-s training sessions. (**c**) Mean (+*SEM*) consumptions of lab chow and the training outcome during the 1-h prefeeding period in the outcome devaluation test. (**d**) Mean ( ± *SEM*) leverpresses in the 3-min extinction test following 1-h prefeeding of lab chow (non-devalued condition) or the training outcome (devalued condition). Group C exhibited selectively decreased leverpresses in the devalued condition compared with the non-devalued condition (*p < 0.05).

Figure 1a summarizes the timeline of the experimental procedure. Food-restricted male Sprague Dawley rats were allocated to one of the three groups with distinct periods of instrumental training based on a random interval (RI) 60-s reinforcement schedule, with 2 (Group A), 8 (Group B), and 16 (Group C) daily sessions. In the RI 60-s schedule, the leverpresses made by the animals were reinforced by the delivery of a food pellet at a rate of one pellet per 60 s on average, which simulated the behavioral adaptation to periodical fluctuations for available resources, because most natural rewards are consumed immediately, but they can be replenished timely. The rats in Group C were subjected to further training for 15 days (extended training), with up to 31 days in total (Group C’). We conducted the experiment using two independent cohorts. The response at the end of acquisition did not differ significantly between the cohorts. According to the two-way analysis of variance (ANOVA) for the leverpress rates by Groups A–C recorded in the last sessions, i.e., *group* (3: A–C) × *cohort* (2), there was a significant main effect of the *group (F*_(2, 26)_ = 14.91; *p* < 0.0001; *η*_*p*_^2^ = 0.53), but the main effect of cohort and interaction was not significant (*F*s < 1.70; *p* ≥ 0.20; *η*_*p*_^2^s ≤ 0.12). One-way ANOVA for Groups C’ (session 31) with *cohort* as the factor also failed to detect any significant main effect (*F*_(1, 8)_ < 1; *η*^2^ = 0.02). Thus, the two cohorts were combined into a single group for subsequent analyses.

Figure 1b shows the learning progress as the changes in the leverpress rates in Groups A–C and C’. One-way repeated measure ANOVA for each group with *session* as the variable detected significant main effects in Group B (*F*_(7, 70)_ = 4.49; *p* < 0.001; *η*^2^ = 0.07) and Group C (*F*_(15, 135)_ = 10.03; *p* < 0.0001; *η*^2^ = 0.29), but not in Group A (*F*_(1, 10)_ = 2.94; *η*^2^ = 0.04). These results indicate that the learning effect was already evident after two RI 60-s sessions and that the leverpress rate continued to increase after eight training sessions. In addition, a significant effect of *session* was detected for the 15 extended training sessions by Group C’ (*F*_(14, 126)_ = 4.04; *p* < 0.0001; *η*^2^ = 0.03). Two-way ANOVA based on the first two sessions of Groups A–C, i.e., *group* (3) × *session* (2), only found the significant main effect of *session (F*_(1, 29)_ = 12.60; *p* < 0.005; *η*_*p*_^2^ = 0.30). One-way ANOVA for the last sessions by Groups A–C found a significant main effect of *group (F*_(2, 29)_ = 11.39; *p* < 0.0005; *η*^2^ = 0.44), and post-hoc tests indicated that the leverpress rate in Group C was greater than those in Groups A (*t*_(29)_ = 4.57; *p* < 0.0001; *r* = 0.65) and B (*t*_(29)_ = 3.44; *p* < 0.005; *r* = 0.54), which did not differ (*t*_(29)_ = 1.21; *r* = 0.22). In addition, one-way repeated measure ANOVA found a significant difference between the last sessions by Groups C and C’ (session 16 and 31, respectively) (*F*_(1, 9)_ = 6.47; *p* < 0.05; *η*^2^ = 0.06). Thus, we conclude that the rate of leverpress increased gradually between three distinct time points from day 8 to day 16, and to day 31.

### Outcome devaluation effect was maximized after a moderate amount of training but not after both extended and limited training

Following the RI training, we assessed the sensitivity of the leverpresses made by rats to the devaluation of the food reward by using outcome-specific satiation^11^. Figure 1c compares the food intake rates in the devalued and non-devalued conditions for the four groups. Two-way ANOVA, i.e., *group* (3: A–C) × *food* (2: lab chow vs. training outcome), and one-way ANOVA for Group C’ with *food* as a factor, only detected a significant main effect of *food (F*_(1, 29)_ = 13.00 and *F*_(1, 9)_ = 11.50; *p* < 0.005 and < 0.01; *η*_*p*_^2^ = 0.31 and *η*^2^ = 0.36, respectively). Thus, the differences in food consumption or motivation across groups did not account for the cumulative effect of training. Figure 1d shows the results of the extinction tests in 1-min blocks. Three-way ANOVA, i.e., *group* (3: A–C) × *condition* (2: non-devalued vs. devalued) × *time-block* (3), detected a significant interaction of *group* × *condition (F*_(2, 29)_ = 5.00; *p* < 0.05; *η*_*p*_^2^ = 0.26), as well as significant main effects of *group* and *condition (F*s ≥ 6.08; *η*_*p*_^2^s ≥ 0.23). The simple main effect of *condition* was significant only in the animals that underwent 16 training sessions (Group C: *F*_(1, 29)_ = 17.92; *p* < 0.0005; *η*_*p*_^2^ = 0.38), and thus, the animals tested following the two (Group A) and eight (B) training sessions did not demonstrate significant devaluation effects (*F*s_(1, 29)_ < 1; *p*s = 0.99 and 0.38; *η*_*p*_^2^s < 0.01 and = 0.03, respectively). The simple main effect of *group* was significant in the non-devalued but not in the devalued condition (*F*s_(2, 58)_ = 10.78 and 0.25; *p*s < 0.0001 and = 0.78; *η*_*p*_^2^s = 0.27 and 0.01, respectively). Two-way repeated measures ANOVA for Group C’, i.e., *condition* × *time-block*, failed to detect any significant main effects (*condition: F*_(1, 9)_ = 2.11; *p* = 0.18; *η*_*p*_^2^ = 0.19, *time-block: F*_(2, 18)_ < 1; *p* = 0.81; *η*_*p*_^2^ = 0.02) or interaction (*F*_(2, 18)_ < 1; *p* = 0.62; *η*_*p*_^2^ = 0.05). These results suggest that the authentic criterion for goal-directed action was applicable only to Group C.

### Selection between trained and untrained levers was balanced in the earliest stage of instrumental training

**Figure 2:**
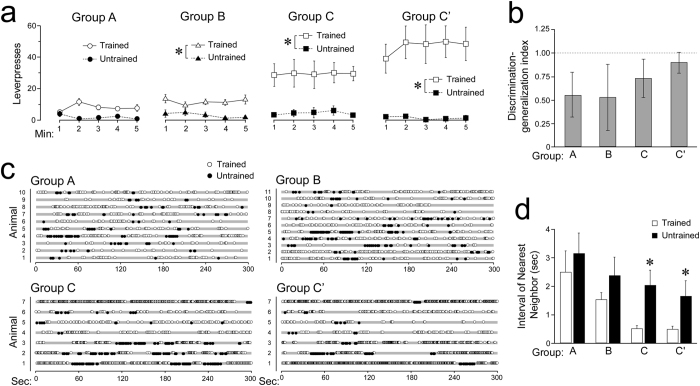
Response balance between the trained and untrained levers changed when instrumental training was repeated. (**a**) Mean (±*SEM*) leverpresses during the 5-min two-lever test where animals were presented simultaneously with both trained and untrained levers (Group A, *n* = 11; Group B, *n* = 11; Group C, *n* = 10; and group C’, *n* = 10). Groups B, C, and C’ exhibited significant performance bias toward the trained lever in terms of the leverpress rate (*p < 0.05), whereas Group A did not. (**b**) The mean discrimination–generalization index was calculated for each group based on the scores shown in box a. Discrimination–generalization index = (X−Y)/(X + Y), where X and Y are the trained and untrained leverpresses, respectively. The 95% confidential intervals of the means are also shown. (**c**) Temporal plots of leverpresses by each animal for both the trained and untrained levers in the two-lever test (Group A, *n* = 10; Group B, *n* = 11; Group C, *n* = 7; and Group C’, *n* = 7). **(d)** According to the temporal plots in box c, we determined the time intervals for the nearest neighbor of each leverpress. For each rat, the median of the intervals was calculated and the group means (+*SEM*) of the individual median scores are shown separately. In Groups C and C’, the interval to the nearest neighbor of the untrained lever was significantly larger than that of the trained lever (*p < 0.05).

Response generalization and discrimination might reflect habitual response and goal-directed action, respectively^11^,^12^. After the devaluation test, animals underwent a two-lever test where the trained and untrained levers were presented simultaneously, and the responses to each lever were compared for 5 min without prefeeding or reinforcement. Figure 2a shows the numbers of leverpresses in 1-min blocks. Three-way ANOVA, i.e., *group* (3: A–C) × *lever* (2: trained vs. untrained) × *time-block* (5), detected a significant interaction of *group* × *lever (F*_(2, 29)_ = 9.16; *p* < 0.001; *η*_*p*_^2^ = 0.39), as well as significant main effects of the two factors (*F*s ≥ 15.39; *η*_*p*_^2^s ≥ 0.51). The simple main effect of *lever* was significant for both Groups B and C (*F*s_(1, 29)_ = 4.69 and 50.40; *p*s < 0.05 and 0.0001; *η*_*p*_^2^s = 0.14 and 0.63, respectively), but not for Group A (*F*_(1, 29)_ = 2.55; *p* = 0.12; *η*_*p*_^2^ = 0.08). The simple main effect of *group* was significant in trained but not in untrained lever (*F*s_(2, 58)_ = 23.80 and 0.22; *p*s < 0.0001 and = 0.81; *η*_*p*_^2^s = 0.45 and 0.01, respectively). To evaluate the effect of extended training on the test performance, we conducted a two-way repeated measures ANOVA using the Group C’ data, i.e., *lever* × *time-block*, which only detected a significant main effect of *lever (F*_(1, 9)_ = 22.93; *p* < 0.001; *η*_*p*_^2^ = 0.72). These analyses indicate that only the rats in Group A, which underwent two RI training sessions, performed the trained and untrained leverpresses without distinction.

We also calculated a discrimination–generalization index for each animal, (X − Y)/(X + Y), where X and Y are the trained and untrained leverpresses, respectively, recorded during the 5-min test. Figure 2b shows the group means and their 95% confidential intervals corrected using Bonferroni’s method (*α* = 0.05/4). According to this index, none of the groups exhibited complete generalization (0.0), but only Group C’ reached the level of perfect discrimination (1.0).

### Generalization of the temporal pattern of the trained leverpresses to the untrained ones changed as a function of the training period

Figure 2c shows temporal plots of the leverpresses recorded for each animal during the 5-min two-lever test. At the initial stage of the training (Group A), both the trained and untrained leverpresses appeared to be sporadic and indifferent to the adjacent leverpresses; however, as the training progressed, the trained leverpresses appeared to be chunked spontaneously (Groups C and C’) whereas the untrained ones did not. To quantify this observation, we employed the time interval median of the nearest neighbor for each leverpress as an index of leverpress contiguity. Figure 2d shows the mean (+*SEM*) of the medians of each group. Two-way ANOVA, i.e., *group* (3: A–C) × *lever* (2: trained vs. untrained), failed to detect any significant main effects (*group: F*_(2, 25)_ = 2.66; *p* = 0.09; *η*_*p*_^2^ = 0.18, *lever: F*_(1, 25)_ = 4.09; *p* = 0.054; *η*_*p*_^2^ = 0.14) or interactions (*F*_(2, 25)_ < 1; *η*_*p*_^2^ = 0.02). To evaluate the effect of extended training on this measure, we conducted two-way repeated measures ANOVA, i.e., *group* (2: C vs. C’) × *lever* (2: trained vs. untrained), which showed that only the effect of *lever* was significant (*F*_(1, 6)_ = 8.26; *p* < 0.05; *η*_*p*_^2^ = 0.58).

## Discussion

In this study, we demonstrated that devaluing the instrumental outcome after a moderate training reduced the leverpress activity (Group C, 16 RI training sessions, Fig. 1d), thereby revealing that the leverpress was a goal-directed action. When the rats in Group C were presented simultaneously with two levers, namely, trained and untrained, they preferred to press the lever they had been trained with (Fig. 2a). Furthermore, although their presses of levers they had been trained with were chunked spontaneously, the presses of the unfamiliar levers remained sporadic (Fig. 2d). These findings suggest that at the stage of goal-directed action, action selection was biased toward the relevant behavioral option rather than the unfamiliar option.

Insensitivity to outcome devaluation appeared after extensive training (Group C’, 31 training sessions, Fig. 1d), as previously reported^2^,^3^,^9^,^10^,^12^. However, in this study the devaluation insensitivity was also found after limited training sessions (Groups A and B, which received two and eight sessions, respectively). In addition, the initial and late outcome devaluation-insensitive stages were distinguished using the two-lever test. Animals in Group C’ reliably discriminated between the levers they had been trained with and other unfamiliar levers (Fig. 2a,b), but they also exhibited trained lever-specific spontaneous chunking (Fig. 2d); however, in contrast, based on all measures, there was no significant difference between the levers they had been trained with and other unfamiliar levers for Group A.

Further analyses of the performance in the two-lever test revealed a difference between the two outcome devaluation-insensitive groups in the initial stage of training, namely, Groups A and B. According to the leverpress rate, the rats in Group A responded to both levers irrespective of their reinforcement history; however, those in Group B reliably discriminated between the levers (Fig. 2a). Nevertheless, in both groups, there was no significant difference between the two levers in terms of the leverpress contiguity (Fig. 2d), which suggests that the animals in Group B employed a generalized action strategy to distinct levers, despite of their ability to discriminate between relevant and irrelevant levers.

Therefore, a habitual response established after the extended training could be segregated with a highly biased action strategy (preferential selection and chunking) as well as insensitivity to outcome devaluation. The instrumental behavior before goal-directed action is also resistant to outcome devaluation, but it could be distinctively characterized by an unbiased action strategy or behavioral generalization, even to an unfamiliar behavioral option. In general, instrumental behavior at the earlier stage of training can meet the criteria for “exploration”^13^,^14^; however, exploration does not necessarily emerge only at the initial stage of reinforcement learning. Indeed, even after habit formation, an agent sometimes suddenly encounters an uncertain situation^15^,^16^ where the efficacy of the previously learned behavior is unknown. For example, during reversal learning or contingency degradation, exploration begins to substitute for the now inappropriate habitual response in order to adapt to the novel situation^13^,^14^. Uncertainty diminishes as a consequence of action–outcome learning; hence exploration may first switch to goal–directed and then to habitual response, which is probably consistent with the reduced firing of neurons in the locus coeruleus^17^. Therefore, generalized responses to untrained behavioral options at the initial stage of instrumental learning might be more reasonable than a strong bias toward trained options because at this stage, the action–outcome contingency could still be too anecdotal and erroneous to pursue an exploitative approach. In addition to the abovementioned explorative state, other possibilities, such as the involvement of associative learning including the “context” in which learning occurs, could account for the behavior^18^ because action–outcome and stimulus–response learning are not sufficiently mature at this time. This factor might have a distinct effect on sensitivity to outcome devaluation and response generalization.

Finally, we note that previous studies have demonstrated reliable sensitivity of instrumental performance to outcome devaluation after a very limited number of sessions compared with that used in this study^19^,^20^. In addition, studies have shown that mice trained under an RI schedule as employed in the present study became insensitive to outcome devaluation and responded to both trained and untrained (unfamiliar) levers irrespective of their reinforcement history^11^,^12^. These observations have been widely accepted as hallmarks during the process from goal-directed to habitual actions in instrumental learning but, unexpectedly, do not appear to be consistent with our findings. One idea to reconcile this discrepancy is that most previous studies employed pigmented strains of rats (e.g., Long-Evans or Lister) or mice (C57BL/6); thus, the use of albino rats in this study (Sprague Dawley) may account for the slower progress of instrumental learning. Notably, no previous investigation compared the taste aversion method employed in previous studies and the satiety method employed in the present study to determine with which method a habitual response is more readily developed. Hence, the methodological differences may have affected the delay in habit formation. In addition, in most previous studies the training phase was completed in 7–8 days, but we found that the transition from “generalization” in the initial stage to “predilection” in the late stage of a learning process extended over a long period of time, which might provide a favorable condition for determining the coexistence of habitual response and response discrimination. Moreover, generalization in the initial and late stages might differ in terms of functional significance. Besides, Sprague Dawley rats, as slow learners, might be beneficial in providing a finer time resolution to monitor any sequential shift/process in behavioral components over sessions.

In the present study, the appetitive instrumental learning exhibited at least two major mode transitions before forming a habit: from an unbiased, generalized response stage (exploration) to goal-directed action, and then to a habitual response. These three distinct behavioral modes were dissociable using the combination of outcome devaluation and tests involving a choice between a lever with which they had been trained and a lever that they were unfamiliar with. These observations may be still regarded as exceptional; however, considering the generalization provided by additional studies, one should be cautious when interpreting the habit-like phenotype observed during instrumental training in a limited number of sessions, when relying only on an outcome devaluation test, under certain conditions.

## Materials and Methods

### Animals and apparatus

Sprague–Dawley male rats weighing 275–300 g (Japan SLC, Hamamatsu, Japan) were housed individually and were maintained under a 12-h light–dark cycle (lights on at 08:45 h). All procedures were conducted in accordance with the Guidelines for Proper Conduct of Animal Experiments (Science Council of Japan, June 2006) and were approved by the Institutional Animal Care and Use Committee of Kanazawa University. Behavioral training and testing were conducted in standard operant chambers (Med Associates, St. Albans, VT, USA). Each chamber was equipped with a recessed food magazine where 45-mg food pellets (F0021; Bioserv, Flemington, NJ, USA) were delivered as a reinforcing outcome, and with two retractable levers on each side of the magazine.

### Procedure

#### Instrumental training

The experiment was conducted in two separate cohorts with local control (cohort 1, *n* = 17; cohort 2, *n* = 15). Animals were handled daily for 5 days and restricted to approximately 12.5 g of lab chow/day (CRF-1; Charles River Laboratories Japan, Yokohama, Japan) to maintain the body weight at 85% of their *ad libitum* weight. Rats were then assigned randomly to three groups; Group A (cohort 1, *n* = 5; cohort 2, *n* = 6; total, *n* = 11), B (same as above; *n* = 6, *n* = 5, and *n* = 11, respectively), and C (same as above; *n* = 6, *n* = 4, and *n* = 10, respectively), which subsequently underwent 2, 8, and 16 daily training sessions reinforced based on an RI 60-s schedule, respectively. The rats in Group C’ were identical to the Group C animals, but they were subjected to additional training for 15 days (extended training), with up to 31 days in total. To ensure that the first outcome devaluation and two-lever tests were conducted on the same calendar days for all animals, Group C began the subsequent training procedure 8 days before Group B, and 14 days before Group A. Initial training comprised five daily sessions: apparatus habituation (30 min), magazine training (30 min where outcome pellets were delivered at a random time 30-s schedule), manual shaping with one lever (right/left counterbalanced across animals with 60 outcomes/30 min), continuous reinforcement training (60 outcomes/30 min), and RI 30-s schedule training (30 min). Leverpressing was then reinforced under an RI 60-s schedule for 30 min each day. The algorithm used to generate these schedules was described previously^21^, where the interval (30 or 60 s) was defined as the ratio between *T* (renewal cycle: 3 or 6 s, respectively) and *p* (constant probability of reinforcement for the first leverpress in each renewal cycle: 0.1). Each session during training and testing started with lever(s) presentation after 300 s of waiting period without stimuli or lever presentations.

#### Outcome devaluation and two-lever (trained vs. untrained) tests

Sensitivity to outcome devaluation was determined in a 2-day test under devalued and non-devalued conditions (order counterbalanced). For the devalued condition, each animal was given 1-h *ad libitum* access to the training outcome in individual consumption cages. For the non-devalued condition, each rat was given 1-h *ad libitum* access to lab chow. Immediately after these satiety manipulations, rats were placed in the operant chambers and their responses to the trained lever were tested for 3 min without food delivery ( = extinction). On the next day of the devaluation test, leverpressing by the rats was monitored in a 5-min extinction test without previous *ad libitum* feeding. In this test, two levers were presented, one for which the rats were trained and another for which they did not receive training (unfamiliar lever). After completing the first devaluation and two-lever tests, the animals in Group C underwent 15 additional RI 60-s training sessions followed by second outcome devaluation and two-lever tests, which were conducted in the same manner as the first set (Fig. 1a).

### Data analyses and statistical tests

According to the temporal plots for trained and untrained levers of each animal recorded in the two-lever test (Fig. 2c), time intervals were recorded for the nearest neighbor for each leverpress. In this analysis, the data for the animals that made 0 or 1 leverpress during testing were discarded because the time intervals to the nearest neighbor could not be calculated. The number of animals in each group after the change is described in the Fig. 2 legend. The median (a measure of the central tendency of samples that is not affected by their distribution) of the intervals was then calculated for each animal, and statistical analyses were performed using the median scores.

In ANOVA tests for the data obtained from the outcome devaluation test and two-lever test, a between-subjects variable (Groups A, B, and C) was used as well as within-subject factors (non-devalued vs. devalued/trained vs. untrained lever, and 1-min time interval), and a within-subject design was employed to assess the data for Group C’. The reliability of the results was assessed against a type I error (*α*) of 0.05. In addition, the effect size was calculated and reported as *η*^2^, *SS*_effect_/*SS*_total_, for one-way ANOVA tests; *r*, √(*t*^2^/(*t*^2^ + *df* ), for post-hoc *t*-tests; and partial *η*^2^ (*η*_*p*_^2^), *SS*_effect_/(*SS*_effect_ + *SS*_error_), for both multidimensional ANOVA and subsequent simple-main effect tests.

## Additional Information

**How to cite this article:** Iguchi, Y. *et al*. Identification of an unconventional process of instrumental learning characteristically initiated with outcome devaluation-insensitivity and generalized action selection. *Sci. Rep.* **7**, 43307; doi: 10.1038/srep43307 (2017).

**Publisher's note:** Springer Nature remains neutral with regard to jurisdictional claims in published maps and institutional affiliations.

## References

[b1] Balleine B. W., Delgado M. R. & Hikosaka O. The role of the dorsal striatum in reward and decision-making. J. Neurosci. 27, 8161–8165 (2007). 17670959 10.1523/JNEUROSCI.1554-07.2007 PMC6673072

[b2] Balleine B. W. & O’Doherty J. P. Human and rodent homologies in action control: corticostriatal determinants of goal-directed and habitual action. Neuropsychopharmacology 35, 48–69 (2010). 19776734 10.1038/npp.2009.131 PMC3055420

[b3] Dolan R. J. & Dayan P. Goals and Habits in the Brain. Neuron 80, 312–325 (2013). 24139036 10.1016/j.neuron.2013.09.007 PMC3807793

[b4] Colwill R. M. & Rescorla R. A. Postconditioning devaluation of a reinforcer affects instrumental responding. J. Exp. Psychol. Anim. Behav. Process. 11, 120 (1985).

[b5] Hammond L. J. The effect of contingency upon the appetitive conditioning of free-operant behavior. J. Exp. Anal. Behav. 34, 297–304 (1980). 16812191 10.1901/jeab.1980.34-297 PMC1333008

[b6] Dezfouli A., Lingawi N. W. & Balleine B. W. Habits as action sequences: hierarchical action control and changes in outcome value. Philos. Trans. R. Soc. Lond. B Biol. Sci. 369 (2014). 10.1098/rstb.2013.0482 PMC4186235 25267824

[b7] Dickinson A. Actions and habits: the development of behavioural autonomy. Philos. Trans. R. Soc. Lond. B Biol. Sci. 308, 67–78 (1985).

[b8] Dickinson A. Omission learning after instrumental pretraining. Q. J. Exp. Psychol. B 51, 271–286 (1998).

[b9] Yin H. H. & Knowlton B. J. The role of the basal hganglia in habit formation. Nat. Rev. Neurosci. 7, 464–476 (2006). 16715055 10.1038/nrn1919

[b10] Dickinson A. . Motivational control after extended instrumental training. Anim. Learn. Behav. 23, 197–206 (1995).

[b11] Hilario M. R., Clouse E., Yin H. H. & Costa R. M. Endocannabinoid signaling is critical for habit formation. Front. Integr. Neurosci. 1, 6 (2007). 18958234 10.3389/neuro.07.006.2007 PMC2526012

[b12] Hilario M. R. & Costa R. M. High on habits. Front. Neurosci. 2, 208–217 (2008). 19225594 10.3389/neuro.01.030.2008 PMC2622741

[b13] Cohen J. D., McClure S. M. & Yu A. J. Should I stay or should I go? How the human brain manages the trade-off between exploitation and exploration. Philos. Trans. R. Soc. Lond. B Biol. Sci. 362, 933–942 (2007). 17395573 10.1098/rstb.2007.2098 PMC2430007

[b14] Daw N. D. . Cortical substrates for exploratory decisions in humans. Nature 441, 876–879 (2006). 16778890 10.1038/nature04766 PMC2635947

[b15] Yu A. J. & Dayan P. Uncertainty, neuromodulation, and attention. Neuron 46, 681–692 (2005). 15944135 10.1016/j.neuron.2005.04.026

[b16] Tervo D. G. . Behavoral variability through stochastic choice and its gating by anterior cingulate cortex. Cell 159, 21–32 (2014). 25259917 10.1016/j.cell.2014.08.037

[b17] Amemiya S. . Noradrenergic modulation of vicarious trial-and-error behavior during a spatial decision-making task in rats. Neuroscience 265, 291–301 (2014). 24480363 10.1016/j.neuroscience.2014.01.031

[b18] Gershman S. J. Context-dependent learning and causal structure. Psychon. Bull. Rev. (2016). 10.3758/s13423-016-1110-x 27418259

[b19] Holland P. C. Relations between Pavlovian-instrumental transfer and reinforcer devaluation. J. Exp. Psychol. Anim. Behav. Process. 30, 104–117 (2004). 15078120 10.1037/0097-7403.30.2.104

[b20] Killcross S. & Coutureau E. Coordination of actions and habits in the medial prefrontal cortex of rats. Cereb. Cortex 13, 400–408 (2003). 12631569 10.1093/cercor/13.4.400

[b21] Farmer J. Properties of behavior under random interval reinforcement schedules. J. Exp. Anal. Behav. 6, 607–616 (1963). 14055961 10.1901/jeab.1963.6-607 PMC1404433

